# Lived experiences, coping strategies, and relationship dynamics of women and men following miscarriage in Kampala district

**DOI:** 10.3389/fpubh.2026.1845108

**Published:** 2026-07-14

**Authors:** Brenda Nalugo, Beatrice Tondo, Simon Kizito

**Affiliations:** Department of Mental Health and Community Psychology, School of Psychology, College of Humanities and Social Sciences, Makerere University, Kampala, Uganda

**Keywords:** coping strategies, lived experiences, miscarriage, phenomenological, relationship dynamics

## Abstract

Miscarriage is a prevalent and distressing experience that profoundly impacts both women’s and men’s psychological wellbeing and relationship dynamics. This study investigated the lived experiences, coping strategies, and relationship dynamics of women and men following miscarriage in Kampala district. The study used a qualitative approach, particularly a phenomenological research design. It employed a purposive sampling technique to select 12 participants who had experienced at least one miscarriage at least 1 month before data collection. The sample included women aged 24–38 years and men aged 28–50 years. Data were collected using in-depth interviews and analyzed following Virginia Braun and Victoria Clarke’s six-phase approach to thematic analysis. The study findings showed that the lived experiences of participants were characterized by an emotional rollercoaster involving denial and sadness, trauma, and a loss of identity. Participant experiences were also marked by a search for an explanation and the pressure to align grief with cultural gender expectations. To navigate this aftermath, participants’ coping strategies included mutual partner support, religion, the pursuit of a replacement baby, and the presence of other children. Furthermore, the experience transformed relationship dynamics; while some couples figured it out together, strengthening their bond, others faced emotional distance and a damaging blame game, outcomes often influenced by the state of the relationship prior to the loss. The study findings highlighted the psychological, social, spiritual, and emotional impact of miscarriage for both women and men. Therefore, there is a need for comprehensive and culturally sensitive support for individuals navigating the aftermath of miscarriage.

## Background

Pregnancy is a deeply emotional experience, often considered a great accomplishment (1). Unfortunately, miscarriages are common, with some women experiencing more than one miscarriage in a lifetime (2). Globally, 20% of pregnancies end in miscarriage, and in Uganda, one-third of women experience at least one miscarriage (3, 4). Miscarriages often happen unexpectedly and suddenly, leaving many questions about the loss, which makes healing difficult (5). The period after miscarriage is challenging for many people and presents significant psychological and relationship difficulties (6).

Globally, the timeline used to classify pregnancy loss as a miscarriage differs. The general timeline for a miscarriage is defined as the loss of a fetus before 20–28 weeks of pregnancy (2). The World Health Organization’s timeline for miscarriage is loss before the 24th week of pregnancy (62).

Miscarriages can have significant psychological consequences for both women and men (7). The psychological effects experienced by men and women are generally similar, with some differences that may be due to factors such as gender and socio-cultural expectations; however, the experiences for both are considered significant (8). The experience of losing a desired pregnancy can result in a range of emotional responses, including feelings of guilt, grief, and depression, both in the immediate aftermath and in the long term (9). A decline in psychological functioning is evident following miscarriage due to the intense emotional reaction to the traumatic experience (10, 11). After miscarriages, expected responses differ and tend to align with internalized gender roles (12, 13). Men often assume a supportive and comforting role and may suppress their grief to be strong for their partner following a miscarriage (14). On the other hand, some women tend to avoid getting pregnant, while others cope by preparing themselves for the worst case scenarios in subsequent pregnancies (15, 16).

Along with individual coping strategies, people tend to rely on their partners to navigate the shared experience of miscarriage and its consequences (17, 18). The way individuals cope with miscarriage affects their relationship dynamics. Some feel closer to their partners, while others are separated by the occurrence of a miscarriage. Even though individuals in an intimate relationship are dealing with the same loss, each partner copes differently, and this changes the relationship dynamics (19, 20). According to Swanson et al. (21), individuals in a relationship can have different reactions to experiencing a miscarriage. (22, 23) number of individuals, especially those in a cohabiting relationship (63).

The present study aimed to explore the lived experiences, coping strategies, and relationship dynamics of women and men following miscarriage in Kampala district. The research questions were as follows: (1) What are the lived experiences of women and men following miscarriage? (2) What coping strategies are used following miscarriage? (3) How does the experience of miscarriage affect relationship dynamics?

## Methods

### Study design

Using a phenomenological research design, the study explored the lived experiences, coping strategies, and relationship dynamics of women and men following miscarriage (59).

### Study setting

The study targeted individuals residing within Kampala district and receiving or having received post-miscarriage care/treatment from Kawempe National Referral Hospital.

### Sample size and sampling technique

The participants included in the study were women aged 24–38 years and men aged 28–50 years. Some had experienced a single miscarriage, while others had experienced multiple miscarriages. A sample of 12 participants (six men and six women) was included using non-probability sampling, particularly purposive sampling (61). Purposive sampling was used to select a sample that fit the study’s inclusion and exclusion criteria. The sample size of 12 was included in accordance with the principle of data saturation (24). Individuals were contacted to explain the purpose and objectives of the study. Interviews and data analysis were carried out concurrently, and by the 12th interview, no new codes had emerged; therefore, data collection was discontinued as data saturation had been reached.

### Inclusion and exclusion criteria

The study was open to women aged 18–45 years and men aged 18–60 years. Individuals whose miscarriages had occurred at least 1 month before contact with the researchers were included. The study included participants who spoke either English, Luganda, or both.

Those who did not receive or were not receiving post-miscarriage care/treatment from Kawempe National Referral Hospital were excluded from the study. Additionally, individuals who had a miscarriage without prior knowledge of their pregnancy were excluded. Those who had experienced a miscarriage less than 1 month before contact with the researchers were also excluded from the study. Individuals residing outside Kampala district were excluded from the study.

### Ethical considerations

The study was reviewed and approved by the Institutional Review Board (IRB) at Makerere University College of Health Sciences, MAKSHSREC-2023-597. Authorization was received from Kawempe National Referral Hospital, and informed consent was obtained from participants before the start of the study interview process.

### Instruments and measures

Data collection was carried out through in-depth interviews. An interview guide was used to conduct in-depth interviews about the lived experiences, coping strategies, and relationship dynamics of women and men following miscarriage. The guide included a list of open-ended questions and prompts relevant to the research questions, which elicited rich, detailed information from the participants about their experiences and perspectives related to the phenomenon of miscarriage. The interview guide included questions such as, *“Can you tell me a bit about your experience of having a miscarriage, whatever you feel comfortable telling me?”* The in-depth interviews were conducted face-to-face to allow the researchers to observe non-verbal cues and body language, which can provide additional insights into the participants’ experiences. The researchers also used audio recordings for the interviews.

### Data analysis

The transcribed interviews were analyzed to identify patterns and themes. Thematic analysis was used in the data analysis process. The thematic analysis was based on Virginia Braun and Victoria Clarke’s six-phase approach to coding and theme development (25).

### Findings

The study included 12 participants, including 6 male and 6 female participants, representing 50% female and 50% male participants. The age range of the participants was 24–50 years. The majority of the participants (75%) were married according to Ugandan legal standards, while 25% were cohabiting. It is important to note that even participants who were cohabiting referred to their partners as husband/wife (Table 1).

**Table 1:** Participant demographics.

Participant identifier	Age	Sex	Marital status	Number of miscarriages	Time since the last miscarriage (months)
P01.1	28	Male	Married	1	10
P01.2	34	Male	Cohabiting	1	13
P01.3	30	Male	Married	2	3
P01.4	36	Male	Married	2	8
P01.5	50	Male	Cohabiting	3	1
P01.6	29	Male	Married	1	6
P02.1	24	Female	Married	1	10
P02.2	26	Female	Married	1	13
P02.3	38	Female	Cohabiting	3	7
P02.4	33	Female	Married	2	4
P02.5	28	Female	Married	1	2
P02.6	27	Female	Married	1	1

P01, male participants P01.1 to P01.6, while P02, female participants P02.1 to P02.6.

### Research question one: what are the lived experiences of women and men following miscarriage?

The study aimed to understand the experiences of women and men following miscarriage. From the interviews, several themes and sub-themes emerged in response to this research question. The main themes identified were Emotional Rollercoaster, the Search for an Explanation, Planned versus Unplanned Pregnancies, Lost Hope and Shattered Dreams, Trauma, Aligning Reactions with Gender Expectations, and Loss of Identity.

### Emotional distress

An emotional rollercoaster can be described as a whirlwind of feelings that may leave an individual feeling confused, emotionally exhausted, and trapped in a cycle. The suddenness of miscarriage experiences can be likened to an emotional rollercoaster. Participants described going through phases of denial, defeat, and sadness. It is important to note that various emotional reactions/experiences exist. Denial, defeat, and sadness leave someone confused and exhausted.

#### Denial

For some participants, the initial response to the miscarriage was denial—a refusal to accept the doctor’s declaration that they had lost their baby. This denial mirrored the emotional distress; the doctors were saying they had miscarried, but they did not want it to be true. They denied what they had been told and, in some cases, opted to seek more opinions from other health facilities.

For participant P02.1, denial was a strong initial response when she was informed that she had miscarried. She wanted to seek the opinions of other doctors because, at that moment, she could not believe she had lost her baby; she did not want to believe she had miscarried. She said,

*“At that moment, I did not believe it. I thought the scan was wrong….. My baby was okay, I did not believe the doctor there…” (P02.1).*

For participant P01.3, denial was a strong emotion experienced that did not dissipate easily. The participant shared,

*“When she called and said she had miscarried, I refused to believe it. I did not even believe it for a long time. I thought there was a mistake, the doctors were missing something” (P01.3).*

#### Defeat

The realization that the miscarriage was happening or that it had happened, and there was nothing that could be done, brought about feelings of defeat. Witnessing the miscarriage happen, even before the doctors confirmed it, and the knowledge that a miscarriage had happened and realizing that nothing that could be done was simply devastating and caused a feeling of defeat.

For participant P02.4, this was her second miscarriage. The second miscarriage happened in a way similar to the first, making it painfully familiar. Once again, as with the previous miscarriage, she felt there was nothing she could do to stop it. She shared

*“I saw it happen. I was in the bathroom, and there was a lot of blood. I could see it. Like the time before, there was a lot of blood. I knew I had lost my baby. I sat there in the bathroom and just cried because what else could I do?...” (P02.4).*

This feeling of defeat continued for days, weeks, and months following the miscarriage, to the extent that it affected her desire to conceive again. Feeling defeated after her third miscarriage, the patient P02.3 shared that she was done trying because she had decided to stop trying. She had conceived thrice and consecutively; all of which ended in miscarriage.

*“I don’t want to try, I am tired. I have decided that I will take a break. Or maybe I am just not meant to have children anymore. I am tired. Why me?”(P02.3)*

#### Sadness

Sadness following miscarriage was experienced by both women and men, and it began the moment the miscarriage occurs and continues afterward. The sadness extended for a vast amount of time: days, weeks, months, and in some cases, years. Some participants continued to experience sadness whenever they remember the loss. Certain events, such as seeing another woman pregnant, could trigger feelings of sadness in the period that follows, and in that moment, the realization that the baby was lost sets in, as well as the vacuum it leaves, with sadness becoming overwhelming.

*“I cried a lot. I cried day and night. I felt so defeated. I failed to sleep, and I was just crying” (P02.5).*

*“That was my baby, I wanted that child so I was sad. And she was so sad after seeing her that way, crying, it hurt” (P01.1).*

### The search for an explanation

To search for an explanation indicates thinking about the events leading up to a certain experience and trying to understand how it came to be. When people are searching for an explanation, they are trying to understand why something happened, what might have contributed to it, and what could have been done differently.

Following the miscarriage, people begin to search for explanations to help them understand the experience. In the process, some turn to God as a higher power having control over what happened, some partners blame each other, while others turn inward, trying to figure out what they did that may have been a contributing factor to the miscarriage. There are three sub-themes under this theme: the Blame Game, God’s Power/Control, and the “Why Me” Question.

#### The blame game

When something bad or negative happens, people sometimes begin to assign blame. This blame is often directed toward those around them. For some, blame is assigned to their partners, and this back-and-forth is termed “The Blame Game.”

In a bid to find an explanation for why the miscarriage(s) had happened, some partners reported blaming their significant others. Some participants described finding fault in their partners. Additionally, some reported feeling that their partners were blaming them for the miscarriage(s).

For example, one participant described how her partner blamed her and the effect it had on her. She said,

*“There were some changes because he believes I am the one who terminated these pregnancies. I don’t even know how a pregnancy is terminated. I feel bad because he blames me ....I felt so bad, I felt like I was fading away, I felt like I wanted to die, but the doctor comforted me.”(P02.6)*

Another participant also described that she blamed her husband for the miscarriage for some time after it had occurred. She said,

*“For him, he is still normal, but for me, sometimes I feel like dumping him because sometimes I feel like maybe he's the reason for the losses. There were times I was rude to him, and he said that I acted like I was the only one who lost the baby, as if for him, he still had the baby” (P02.2).*

#### God’s power/control

Turning to God was a common sub-theme for nearly all participants. Following the occurrence of a miscarriage(s), many participants reported turning to God as a higher power in control. In their efforts to make sense of their experience, the resolve that it is all in God’s control was common.

*“Because I am a believer, I just accepted that it’s probably God’s will. God has reasons for everything. So I left it to God” **(**P01.4).*

*“I feel like God has a reason for everything….Me and my husband pray a lot, and we believe it happened because it was God’s will” (P02.2).*

#### The “why me” question

Often used after the occurrence of a negative situation or experience, the question “why me” is used in a bid for people to understand why an unfortunate event has happened to them. This question is therefore a state of introspection in which someone wonders why a bad omen has befallen them.

This theme was mostly common among the female participants who, following miscarriage(s), turned inward in search of an explanation for what had happened and why it had occurred. Some participants reported feeling like they were at fault or that they must be at fault for the miscarriage experience.

*“I felt so bad, I was really discouraged because why me? The first one felt normal, and then I lost the baby, and then this one, I just felt so bad, I cried. I felt like dying even. Honestly, I am fed up even” (P02.2).*

*“Many thoughts, I kept asking why me, why has this happened. Because I spent two years wanting a child, but I had failed to conceive. I felt so much pain and still, I feel so much pain because I have still failed to conceive, so I feel defeated” (P02.5).*

### Planned vs. unplanned pregnancies

The impact of a loss is, in some cases, not dependent on whether it was a planned pregnancy or not. Additionally, the conception process comes into play when a miscarriage happens. If the conception process was difficult, the impact of the miscarriage can be seen to differ from when conception was easy.

One male participant whose partner had a miscarriage described how having children prior to the miscarriage affected the impact of the miscarriage.

“*...Getting kids was never a priority because I already have kids ... .The pregnancy is not really a priority for me; I didn’t even want the children, but if she wants then she can have them because I already have 6 children and those were enough” (P01.5).*

A female participant, on the other hand, who had no children before the miscarriage, described her experience. She said,

*“Because I spent two years wanting a child, but I had failed to conceive. I felt so much pain, and still I feel so much pain because I have still failed to conceive, so I feel defeated” (P02.3).*

### Lost Hope and shattered dreams

In this theme, all the hopes and dreams the participants had for the baby they were carrying were shattered when the miscarriage happened. Participants explained how the hopes they had when envisioning the life they would have as parents of the baby they had conceived were all lost the moment the miscarriage happened. One participant shared how the miscarriage left her empty and discouraged because she had imagined a life with her child, and with the miscarriage, that was no longer a possibility. She said,

*“I felt so bad, I was really discouraged because why me? I just feel so empty, I lost my baby. I had started buying things, I had plans. Then the miscarriage happened, so I don’t want to try anymore” (P02.2).*

Multiple miscarriages also cause an extreme loss of hope. Participants described how the recurrence of miscarriage made them begin to question whether they should continue trying to conceive. Their dreams of motherhood were shattered with each miscarriage.

*“I was so scared after this last miscarriage. Had decided to not try because what is the point if it ends up having miscarriages? She cried so much, was in a lot of pain, and I was so helpless” (P01.2).*

*“I am afraid. I am now afraid that another miscarriage will happen…..what if I get pregnant and miscarry again? Before, I really wanted to give birth, but not anymore. I think now, even if I got pregnant again, I would not care. If a miscarriage happens, it happens. I’m just there, I don’t care anymore” (P02.3).*

### Trauma

Trauma refers to severe shock following a bad or upsetting experience, which may cause psychological or emotional damage. The experience of miscarriage can be categorized as traumatic. The experience of miscarriage involves seeing and hearing about a death. Some participants described seeing the miscarriage happen, and in those moments, there was nothing they could do to stop it. The feelings of defeat and helplessness when life is lost can be traumatic. Some describe feeling an emptiness afterward, while others go back and forth between knowing the loss happened and feeling the presence of the baby that was lost. These feelings also stay present and remain impactful for a while. Some participants described feeling afraid because they were unsure about the possibility of the recurrence of miscarriage.

Two participants described witnessing the miscarriage as it happened and experiencing intense physical pain:

*“I saw it. I was in the bathroom when it happened, and I saw it….When the miscarriage was happening, I could tell. At the time of the miscarriage, I felt pain similar to labor pains. I went to the hospital, but still, the miscarriage happened, and I saw it. It scares me up to today” (P02.4).*

*“I could feel the miscarriage happening, I felt it pushing down. It was a really bad experience*” *(P02.6).*

Another participant described living with continuous fear after the miscarriage. They are afraid that another miscarriage might happen.

*“But for me, I don’t want to because even the six months I feel like they are short. I don’t want to conceive for another year because I’m not ready, what if it happens again...Even now, when I think about that time, I just feel like maybe I should wait to get pregnant” (P02.2).*

### Aligning reactions with gender expectations

Gender expectations of reaction pertain to gender-specific reactions to different situations as well as the stereotypically expected reactions of members of either gender in given situations. One of the themes that came up about the experience of miscarriage was the link/affiliation of gender to reaction, participants aligning their reaction to the miscarriage with what is expected of their gender.

Participants reported aligning their reactions with gender expectations. They therefore confined their reactions to the miscarriage to what was culturally expected of members of their gender. Additionally, participants also reported accepting and expecting certain reactions from their partners based on the gender of the latter.

One participant expressed acceptance of her partner’s reaction because of his gender. Because he is a man, she accepted that his becoming distant was the expected reaction. Additionally, she believed that because she is a woman, she has to be strong.

*“From that day, during that time, he changed. But you know, men, for us women, we just stay and keep strong. Of course, I felt bad. I felt bad. It hurt me, but what comforted me, I got to the point of just knowing that men are just that way” (P02.5).*

Another participant shared that, as the one who carried the baby, her experience of the miscarriage was more intense and felt that her husband definitely did not understand her.

*“For me, I was the one carrying, so I don’t think he understood because for me I was the one who could feel the baby, and I even told him this, but he told me he also felt the pain. But we talked, and we are fine now” (P02.2).*

### Loss of identity

Child loss represents more than just the end of a relationship with the deceased; it also signifies the loss of a part of oneself. When people conceive, part of who they are and their identity becomes tied to the possibility of becoming parents. For female participants, the loss also led them to question who they were and whether they were “woman enough.” Participants shared,

*“I felt really bad. As a woman, I feel really bad having all these miscarriages. Am I even woman enough?” (P02.3).*

*“I don’t even know what to do anymore. My husband actually has another woman, and she has children. But I don’t. I don’t even blame him for spending more time with her because am I even woman enough? He has always wanted children, and I have failed to give him any” (P02.4).*

A participant shared how lost he felt after the miscarriage because when his partner told him she was pregnant, he was so excited that he had started telling everyone he was going to be a father and that had become his identity. He shared,

*“I was so excited. I was so ready to be a father; I wanted to be a father. I told everyone, and I was just very happy. And then she called me when I was at work and said she was in the hospital. They said she had lost the baby, and I don’t know, I just sat there, I was so lost” (P01.1).*

### That should be me

This phrase indicates a longing to be in the place of another because one feels as deserving, if not more. The feeling that resorts to this phase, *“That should be me,”* can be likened to feeling robbed of an opportunity, and thus, an individual feels like they should be experiencing, but they are not. This theme describes the experiences of participants who struggled following a miscarriage(s) specifically because, when they looked around them, they noticed other women see carrying their pregnancies to full term and having children.

*“But then I would see the women who got pregnant at the same time as me, and they have babies, and that would hurt me, so I would cry, but then I would turn to God, and He strengthened me” (P02.3).*

*“Some days I look at the women who were pregnant when I conceived, and I cry because they now have babies and I don’t. It hurts” (P02.5).*

### Research question two: what coping strategies are used after a miscarriage?

This research question aimed to gain a comprehensive understanding of the coping strategies employed following a miscarriage(s). From the interviews, the following themes were prevalent: The Partner’s Mutual Support, The Presence of Other Children, Religious Coping, A Replacement Baby, and “It’s not just me.

### The partner’s mutual support

A theme that emerged was the significance of mutual partner support in a relationship. Participants reported that leaning on each other was critical in navigating the emotional turmoil and loss. This theme highlights the dynamics of shared grief and emotional bonding and communication, which are explored below as sub-themes.

#### Shared grief and understanding

Having experienced a miscarriage in a relationship, grief is processed jointly. Partners mourn a shared loss because it was ‘their’ child. There is a unique understanding that exists between partners who have experienced the same loss, an understanding that cannot always be extended outside because grief from loss by miscarriage is disenfranchised. This shared grief created a foundation for mutual support and empathy, which aids coping. Participants shared,

*"He was the only one who truly understood the depth of my pain. We cried together, and that made it bearable." (P02.1).*

*"We didn't need to explain our feelings to each other. Just a look or a hug was enough to convey everything. We had both experienced the loss, so we understood" (P01.1).*

#### Emotional bonding and communication

Open communication, in which the miscarriage is discussed comfortably within the relationship, aids in coping. Talking about their feelings, fears, and hopes helps them process their grief collectively. Participants shared that having conversations about the miscarriage with their partners helped them cope.

*"Talking about what had happened helped us heal together. We shared what we were feeling, and it helped us" (P01.1).*

*"Our relationship grew stronger because we allowed ourselves to be vulnerable with each other. We learned to lean on one another more than ever" (P02.1).*

### The presence of other children

The presence of other children refers to a situation in which one has children together with their partner or outside the relationship. Whether there are other children or not is a factor that comes into play when coping with a miscarriage experience. The presence of other children before the miscarriage seemingly softens its impact. Additionally, couples who have or conceive other children following the miscarriage tend to have a more positive experience in comparison to those who have no children.

One participant shared how her husband used having other children before the miscarriage as a point of comfort to support her and help them cope with the experience. She said,

*“My husband sat with me and was supportive. He said it was all okay because we both have children and we should be happy I am alive because I think if he had stressed me, I would have been in so much pain, but he was supportive” (P02.3).*

Another participant disclosed that because he had children before; hence, the miscarriage experience did not affect him as much. He shared,

*“Me loving her had nothing to do with giving birth because I have my own children… So when the miscarriage happened, I wasn’t affected because pursuing her, my program was not for us to have children, I already have children” (P01.1).*

### Religious coping

Many people turn to their religions to understand the reasons behind their miscarriage, viewing it as part of a divine plan. This coping mechanism involves the use of spiritual beliefs and practices to manage the emotional, psychological, and social stressors that arise following miscarriage. However, religious coping can be either positive or negative, which was the experience for some participants.

#### Positive religious coping

Positive religious coping strategies include seeking spiritual support, reframing the loss as part of a larger, benevolent plan, and finding strength in one’s faith. Turning to religion for positivity was a coping mechanism used after a miscarriage.

For one participant, leaning on religion helped her cope. She shared that she believed God was there for her, which helped her after the miscarriage. She said,

*“Yes, it was all God, God has always been therefore me. Even now, thinking of that time, I want to cry, but I know that God is there, if I pray to Him, He will be there” **(**P02.3).*

Another participant shared that she found comfort in praying to God, which helped her cope with the miscarriage. She shared,

*“I prayed. God helped me. Every time I felt down, I prayed to God, and he helped me” (P02.1).*

#### Negative religious coping

Conversely, some individuals might experience negative religious coping where they feel like their experience is a punishment from a higher power. This can result in feelings of isolation, guilt, and despair.

For some participants, coping came with guilt. They felt that the miscarriage was a punishment from God, and therefore, to cope, they immersed themselves in religious rituals seeking God’s forgiveness. For example, one participant shared,

*“I thought that maybe God was punishing me for something. I accepted what had happened. I prayed and fasted for forgiveness so my baby is blessed the next time I get pregnant” (P02.2).*

### A replacement baby

A replacement baby refers to the child conceived following a miscarriage. Following a miscarriage, some people intentionally get pregnant with the intent to replace the child that they lost.

During the interviews, some people disclosed that they felt it necessary to try and conceive soon after the miscarriage. For those who were able to conceive, the replacement baby allowed them to cope, bringing a form of healing. For example, a participant said,

*“My husband, my parents, my friends- they all supported me. That I will get another baby, they supported me….. And I got another baby, we were so happy. It really helped, having another baby has helped us heal” (P02.1, Female).*

On the other hand, people who fail to conceive after a miscarriage when they desire a replacement baby may have a hard time coping. For example, a participant shared how the failure to conceive soon after a miscarriage stunted her healing:

*“It has been over a year, and only now am I starting to recover. Musawo, I tried to conceive in those first months after, and I failed; it was hard. I couldn’t even get another baby to help fill our hearts, I haven’t even conceived up to now” (P02.3).*

### It’s not just me

The phrase “it’s not just me” can be likened to a sentiment in which one realizes that their experience is not isolated. It denotes the comfort that comes from knowing that there are other people who have been through similar experiences.

With this theme, participants shared how hearing from other women who had experienced miscarriages helped them cope because it put an end to their feelings of alienation. Hearing other people’s experiences helped them heal and cope better. For example, one participant shares,

*“What helped me was seeing that there are so many women with similar cases, and some don’t even have children, so I counselled myself like that because at least I have children.”(P02.4).*

*“My neighbor, who took care of me after, and she had experienced a miscarriage before. She was really comforting. And she has children now, so I know that I will also have children later, I’m just not ready yet”. (P02.2)*

### Research question three: how does the experience of miscarriage affect relationship dynamics?

Experiences such as miscarriage sometimes cause changes in how people relate to each other, and this research question aimed at looking into those changes. Many themes emerged in line with the changes in relationship dynamics after the occurrence of a miscarriage(s), and these were Figuring It Out Together, The State of the Relationship Before Miscarriage, Blame Game, and Emotional Distance.

### Figuring it out together

“Figuring it out together” indicates people working in unison with their partners to find a solution or a way out of a distressing situation. It was evident that how individuals approached the effects of the miscarriage significantly impacted their relationship dynamics. Facing the effects of miscarriage together predicted positivity in the relationship dynamics, while dealing with the effects individually created distance between partners, negatively affecting their relationship.

For example, one participant shared that her husband’s presence and support after the miscarriage made her feel loved, strengthening their relationship. She said,

*“He stayed supporting me. He encouraged me. He even got leave for two weeks and stayed with me when I left the hospital. So for us, we became closer. I could see that he loved me and that made me really happy” (P02.1, Female, 24 years).*

However, although some participants worked things out together, others felt disconnected from the experience. A participant whose partner distanced herself after the miscarriage felt isolated, which negatively impacted their relationship. He said,

*"I don’t know her anymore. After we lost the baby, she stopped telling me things. I wouldn’t even know when she was in pain…She doesn’t want me to touch her. It was too much, so I decided to give her space. Moved into a separate room, but most times I work far away cause I don’t want to go home" (P01.4)*.

### The state of the relationship before miscarriage

This theme explores whether individuals had a loving relationship before a tragic experience, such as a miscarriage, and the implications of that experience. A strong, loving relationship prior to the miscarriage often meant that the love and trust carried over, and their relationship dynamics remained unaffected by the experience. Conversely, in cases where there was mistrust and a lack of love, the miscarriage often worsened the relationship dynamics. Participants indicated that when a loving relationship existed before the miscarriage, relationship dynamics remained poor after miscarriage. When asked about changes in their relationships after the miscarriage, participants with more positive meaning experiences shared,

*"Ahh, nothing. We had no issues before, so we had no issues after. She came back from her parents' home, and I told her that’s part of life, and we moved on. I have always loved my wife" (P01.3*).

*"No, our relationship did not really change. He stayed supporting me. He encouraged me" (P02.1*).

On the other hand, some relationships were already strained, and the miscarriage experience only worsened the situation for them. One participant commented,

*"The dynamics were already bad. You know, women in their first trimester, she was complicated already" (P01.4*).

Another participant expressed suspicions of infidelity before the miscarriage and worsening dynamics afterward. He said,

"*Of course, there were changes. She changed, but me I thought that maybe the pregnancy was not mine…..we were fighting before, so it all got worse" (P01.6).*

### Blame game

When something negative happens, people sometimes begin to assign blame. In the context of miscarriage, this blame is often directed toward one’s partner, creating what is termed the blame game. Individuals who engaged in blaming their partners experienced intense negative feelings and very poor relationship dynamics, while those who refrained from assigning blame had healthier dynamics.

A participant mentioned, *"We were just wondering what might have happened, but there was no answer, so we just stayed strong… Our relationship remained the same, we love each other" (P01.1).*

In contrast, another participant described the damaging effects of blame following the miscarriage. She said,

*"There were some changes because he believes I am the one who terminated these pregnancies….Even right now, even though we are married, we no longer share homes permanently…" (P02.6).*

### Emotional distance

Miscarriage sometimes introduces emotional distance between partners. This theme explores how feelings of emotional distance can affect relationship dynamics, which sometimes lead to a breakdown in communication and intimacy.

A participant shared that, after the miscarriage, she felt distanced from her partner on an emotional level because he seemingly could not understand how the loss was affecting her. She said,

*"I felt so alone after the miscarriage. He didn’t understand why I needed to grieve so deeply, and I felt like I couldn’t reach him anymore" (P02.2).*

Another participant shared that, after the miscarriage, their partner shut them out emotionally, which affected their relationship dynamics. He shared,

*"She shut me out completely. I wanted to be there for her, but she wouldn’t let me in. It was like she was in a different world; our relationship completely changed. We did not communicate, so things became bad" (P01).*

## Discussion

### Lived experiences of women and men following miscarriage

The study aimed to understand the lived experiences of couples after miscarriages through the research question, “What are the lived experiences of women and men following miscarriage?” This section explores the different themes identified in response to this research question, examines related research associated with these themes, and discusses their relevant implications. Some of the themes identified and discussed in this section include the search for an explanation, which is a common phenomenon that occurs after a loss like a miscarriage and was an identified theme in this study when participants were asked about their miscarriage experiences (26). Participants searching for an explanation as to why the miscarriage happened blamed each other for the miscarriage, while some felt that they were being faulted. This fits into the sub-theme, The Blame Game, which aligns with the findings by Omar et al. (27), where a number of participants disclosed that they felt like others blamed them for the miscarriage. The Blame Game most likely comes into play because of the belief that something must have been done by the individuals involved to cause the miscarriage.

The search for an explanation was a significant theme, with participants seeking to understand why the miscarriage had occurred. This quest for meaning is a common response to loss, as individuals try to make sense of their experiences (26). Participants described engaging in the “blame game,” turning to religious beliefs, and introspectively questioning “why me” These responses reflect the diverse ways in which individuals attempt to attribute causality and find solace. The search for an explanation is a common pattern in sudden, unexplainable situations, such as the occurrence of a miscarriage. Because people believe there is a cause for such events, they seek explanations to understand what happened, sometimes going through different lengths, including blaming their partners.

Conversely, turning to religious or spiritual explanations can provide comfort and a sense of acceptance, as participants noted the belief that the miscarriage was part of a divine plan. The “why me” question represents a struggle with self-identity and the fairness of the loss, which is a critical aspect of grieving (28).

God’s Power/Control is a sub-theme that came up for research question one, with participants in the study sharing how they turned to God to make meaning of their miscarriage experience. The study findings align with previous studies that explored the concept of spirituality regarding its role after miscarriage (29, 30). Allahdadian and Irajpour (29) concluded that religion aids in acceptance and recovery after pregnancy loss. We conceptualized that perhaps people turned to God after miscarriage because they believed that He would provide solace for what happened and why. They believe God is superior and in control of everything, including the miscarriage.

Participants shared that they began to ask themselves why the experience had happened to them. They started to question whether they had done something wrong, which aligns with a study by Nikčević and Nicolaides (26), in which the results indicated that people continue to search for explanations after a miscarriage has happened, and many wonder why the experience happened to them. Wanting to know may also be a way for people to gain control to avoid a recurrence of the experience.

The experience of miscarriage has been described as an emotional one. Emotional rollercoaster was a theme that came up in the exploration of the lived experiences of women and men in this study, with participants sharing that they went from feeling joyous over the pregnancy to being sad and feeling hopeless and that they continuously cried and felt defeated after the miscarriage. The theme “emotional rollercoaster,” which emerged in the study, aligns with the findings of a study by Nynas et al. (31), who described the post-miscarriage period as being marked by emotional distress. The emotions associated with the miscarriage often persist even after time has passed and may become more intense around loss anniversaries, during future pregnancies following successful conception, and in cases where conception does not occur (32, 33). Miscarriage is a loss that is grieved, which may explain the intense emotions. Additionally, the emotional rollercoaster may arise because not only is the child lost in the present, but the miscarriage also puts an end to future dreams of parenthood and life with the child.

The theme of the emotional rollercoaster captures the intense and varied emotional responses women and men experience following a miscarriage. The participants’ experiences following a miscarriage align with the Dual Process Model of Coping with Bereavement (34). The findings revealed that participants went through phases of denial, defeat, and sadness, which represent the loss–orientation phase, where individuals focus directly on the circumstances and pain of the loss. These reactions were not experienced as linear stages; rather, participants oscillate between emotional states while simultaneously engaging in restoration–orientation, such as adjusting their relationship dynamics and seeking mutual support. This reaction is consistent with the literature, which suggests that denial serves as a defense mechanism to protect individuals from the immediate shock and pain of the loss (35). They do not want their loss to be real; hence, they refuse to accept the news or the diagnosis of a miscarriage.

The feeling of defeat, highlighted by participants, underscores the helplessness and powerlessness experienced during and after the miscarriage. This finding aligns with existing research indicating that miscarriages often leave individuals to feel a lack of control and agency over their reproductive outcomes (36). The pervasive sadness reported by participants is also well documented in the literature, as grief from miscarriage can be profound and long-lasting (37). Seeing the miscarriage occur, as described by some participants, is likely a factor contributing to the feelings of defeat; they see it happen but cannot do anything about it.

The theme of aligning reactions with gender expectations reveals how societal norms and cultural expectations shape the way individuals process and express grief. Male participants reported feeling the need to be strong and stoic, while female participants felt a deeper personal loss due to their role in carrying the pregnancy. These findings are consistent with gender studies literature, which suggests that men and women often adhere to traditional gender roles when coping with loss (38). Understanding these gendered expectations can help in providing more tailored and sensitive support to both partners. It is likely that, after a miscarriage, individuals continue to feel the need to align their reactions with societal expectations associated with their gender because, even in the face of loss, society still holds expectations of them.

Gender expectations regarding reactions were a theme that emerged when participants were asked about their miscarriage experiences. Participants reported that their reactions were confined to their gender’s cultural expectations. Men seemingly externalized the experience and only processed the psychological implications of the loss when they observed their partners’ reactions (39). Participants also disclosed that they had expectations for how their partners would react to the experience, and these expectations were tied to gender roles. Additionally, the study showed disparities between women and men, with women expressing that they felt the loss impacted them more deeply than it did men because they were the ones carrying the pregnancy, which aligns with the findings of a study by Farren et al. (40). In the study by Farren et al. (40), women were found to have more anxiety and depression symptoms after miscarriage in comparison to their partners.

The experience of miscarriage was described as traumatic by participants, with some witnessing the physical loss and experiencing intense emotional pain. Trauma from miscarriage can result in long-term psychological effects, including post-traumatic stress disorder (40). The visceral nature of the miscarriage experience, coupled with the sudden and uncontrollable nature of the loss, contributes to the trauma described by participants.

The experience left participants feeling scared and instilled a great level of fear, all of which was characterized as traumatic. The experience of trauma regarding miscarriage for the participants of the study aligns with the results of a study about women’s experiences of miscarriage in which participants described the experience as traumatic (41). In contradiction, a study by Farren et al. (40) questioned whether the miscarriage experience qualified for categorization as traumatic.

The findings of the study showed that the motivation to have a baby influenced the impact of the miscarriage on both women and men. The desire for a child, whether planned or unplanned, shaped the emotional and psychological responses to the loss. Participants who had difficulty conceiving or who had strong desires for parenthood reported feeling a deeper sense of loss and despair. This finding is consistent with research that highlights the significant impact of reproductive challenges on mental health and wellbeing (42). The loss of a desired pregnancy has long-lasting and deep psychological consequences (9).

The results of a 2014 study by Robinson align with the results of this study. Participants shared that the miscarriage had a great emotional impact because they had desired the pregnancy, while those who did not desire the pregnancy were seemingly unaffected by the experience. However, results of a study on women who have experienced miscarriage showed that it did not matter whether the pregnancy was initially desired or not; the experience of miscarriage has strong emotional consequences. This can be explained by the understanding of the maternal bond formed between mother and child during pregnancy, which is not dependent on whether the child was planned for or not.

The theme of lost hope captures the profound disappointment and disillusionment people felt after experiencing one or multiple miscarriages. Participants expressed feelings of emptiness and a reluctance to try conceiving again due to fear of repeated loss. This finding aligns with studies that show that recurrent miscarriages can lead to heightened anxiety, depression, and a sense of hopelessness (43). The cumulative impact of multiple losses can erode the hope and optimism that individuals have about their reproductive future.

Loss of identity was a poignant theme, particularly for female participants who questioned their womanhood and self-worth following miscarriages. This theme underscores the deep personal and social implications of reproductive loss. For many women, the ability to conceive and carry a child is closely tied to their sense of identity and fulfillment (44, 45). Additionally, getting pregnant, carrying to full term, and giving birth heightens a woman’s social status as the experience of pregnancy and childbirth is considered a sacred virtue (46, 47). The loss of a pregnancy can therefore trigger a crisis of identity and self-esteem, as women struggle with feelings of inadequacy and failure. This loss of identity can be explained by the societal expectations that a woman reaches fulfillment of her purpose when she becomes a mother; consequently, when a miscarriage happens, some women feel that the fulfillment of purpose is interrupted. Individuals are unequipped to navigate the loss, external social support networks are often unprepared and hesitant to provide meaningful comfort, which leads to isolation, often associated with increased distress and poor coping (60).

### Coping strategies used following miscarriage

This section explores the coping strategies women and men employed after miscarriage in response to the research question “What Coping Strategies Are Used after Miscarriage?” It examines the different themes identified, related research, and any relevant implications. The themes identified included the presence of other children, religious coping, replacement baby, it’s not just me, and partner’s mutual support.

The presence of other children was a theme related to coping. Having other children before the miscarriage helped in coping with the experience, possibly because it offered reassurance that conceiving was still a possibility (48). People may find consolation and comfort in having other children, which help them to cope with the loss, while those with no children may be more vulnerable. Having children appears to offer some protection against feelings of hopelessness and devastation among couples, but it does not completely alleviate the impact of the loss. In her writing following interviews with 27 women who had experienced miscarriages, Cecil (49) noted that while women generally found some solace in their living children, “their existence did not provide an alternative to the desire for a baby” (p. 421). Other studies have shown that the absence of children is linked to higher depression scores and increased grief (50, 51).

Religious coping played a significant role in aiding the majority of the study participants to cope with the miscarriage experience. Turning to their religions and faith was a source of comfort and hope for participants, and for some, it even provided an answer for the miscarriage, which may have been beneficial for them in coping. The benefit of religion in regard to coping may be associated with the social support that religious circles provide (52). According to Allahdadian and Irajpour (29), religious beliefs and practices, regardless of their type, help parents who have experienced pregnancy loss to better cope with and adapt to the devastating effects of their loss. Additionally, miscarriage is a frightening experience for many; when they recover from it, they may turn to religion in gratitude, which facilitates coping as recovery becomes the silver lining (30).

On the contrary, some studies find no evidence that religious participation aids individuals in coping with pregnancy loss (53). Some research indicates that religion can sometimes inhibit the coping process, as negative religious coping is linked to higher levels of grief among women experiencing pregnancy loss (54). Some parents report feeling isolated within their religious communities due to the emphasis on childbearing, which can lead women who miscarry to feel stigmatized and subsequently reduce their religious involvement (55).

Some try to conceive soon after a miscarriage to fill the gap left by the loss, thereby seeking a replacement baby. In the study, a participant (P02.1) shared that having a baby soon after her miscarriage helped her and her partner cope. On the other side, during subsequent pregnancies, feelings of grief and loss can surface while bonding with the unborn child, potentially compromising prenatal attachment. Additionally, unresolved grief, postpartum depression, and postpartum anxiety can profoundly affect postnatal attachment, potentially leading to insecure attachments (56). The idea of a replacement baby is likely a coping mechanism because it allows for the fulfillment of the desire to become parents.

Study results showed that, for those who have experienced miscarriages, finding out that others have similar experiences is comforting, as they begin to feel less isolated with the realization that it’s not just them. Miscarriages are often unspoken about, which increases stigma around the experience (41). Women reported that hearing from other women who have had miscarriages in the past and went on to have children gave them hope for their own future pregnancies (57). It is possible that hearing about similar experiences facilitates coping because it not only demystifies miscarriage but also eliminates the isolation that comes with loss. Additionally, in cases where people with similar experiences went on to have children, sharing and hearing about their experiences becomes a source of hope.

### Relationship dynamics following miscarriage

The study aimed at understanding the changes that the experience of miscarriage(s) caused in relationship dynamics by answering the research question: “How does the experience of miscarriage affect relationship dynamics?” This section explores the different themes identified in response to this research question, related research to the themes, and any relevant implications. Some of the themes that were Figuring It Out Together, The State of the Relationship Before Miscarriage, Blame Game, and Emotional Distance.

For the study, those who worked together as a unit with their partners to deal with the aftermath of the miscarriage showed more positive results in their relationship dynamics. The occurrence of the miscarriage seemingly united them as they joined forces to work through the experience. These results align with the findings of a dyadic study in which couples reported that viewing the miscarriage as a shared loss helped strengthen their relationship (18). A strong sense of commitment and relying on one’s partner as the main source of support enhances emotional wellbeing and strengthens the bond between partners, fostering a greater sense of closeness in the relationship following a miscarriage (58). This may be because partners find comfort and support in each other, and the healing process is shared. On the other hand, those who do not deal with the experience jointly with their partners are likely to experience negative implications for their relationship.

The state of the relationship before the miscarriage was indicative of the relationship dynamics afterward. That is, a loving relationship prior to the miscarriage was a determinant for positive relationship dynamics, according to the results of the study. Participants shared that having love for their partners beforehand helped keep their relationship firm even after the miscarriage, while the opposite was true for those who had relationship problems before the miscarriage. This is most likely because commitment to the relationship creates a sense of unity, which leads to more positive relationship dynamics (18). Where prior commitment, love, and a sense of unity within the relationship existed, the miscarriage was seen as an experience for the partners as a unit, not isolated individuals, which facilitated better relationship dynamics.

Participants shared that feeling blamed by their partners for the miscarriage led to fights and, therefore, negatively affected relationship dynamics. Additionally, participants who faulted their partners for the miscarriage ended up resenting them. Men, in a bid to find an explanation for the miscarriage, questioned whether their partners (the women) who were carrying the pregnancy may have done something to contribute to the pregnancy (13). It does not help that several women already blame themselves for the miscarriage experience or feel like other people around them blame them, so they may self-isolate in guilt, which negatively affects their relationships (9).

Emotional distance emerged as a theme affecting the relationship dynamics of both women and men following miscarriage. The woman who was carrying the pregnancy prior to the loss may feel like she is the only one who feels its impact and thus closes off to her partner, which creates negative changes in relationship dynamics. This emotional distance causes the changes, probably because the partner who distances feels unsupported, while their partner also feels neglected.

### Limitations

The use of purposive sampling, while appropriate for the phenomenological approach, can arguably introduce selection bias as it relies on the researchers’ judgment in selecting participants who fit specific criteria. The study was also geographically limited to those residing in Kampala district, which may not represent the experiences of couples in different cultural or socioeconomic contexts, given how diverse Uganda is. Furthermore, the reliance on self-reported data through in-depth interviews may result in recall bias, as participants might have difficulty accurately recalling their emotions and experiences related to their miscarriage. The study did not explore the difference in experience depending on the timeline since the miscarriage, which limited the extent of the study findings.

### Strengths

The study’s qualitative, phenomenological approach enabled a deep exploration of the experiences of both women and men after miscarriages, thoroughly examining the complex and emotional aspects of the topic. The diverse participant group, balanced between men and women aged 24–50 years and including both married and cohabiting individuals, offered a wide perspective. This diversity enriched the data, providing a detailed understanding of how various individuals and relationships handle miscarriage. The systematic thematic analysis, outlined in the results and discussion sections, identified key themes like the search for explanations, emotional highs and lows, and gender-specific reactions. By comparing these findings with existing research, the study contextualized its results and suggested areas for further investigation, offering valuable insights into clinical psychology and the understanding of miscarriage experiences.

### Recommendations

Healthcare providers should develop and implement comprehensive support services tailored to the needs of individuals who experience miscarriage. These services should include psychological counseling, support groups, and access to resources that address both emotional and relational impacts. Emphasizing the importance of addressing both partners’ experiences can help mitigate feelings of isolation and blame, fostering a shared healing process.

Given the significant role of religion and faith in coping with miscarriage, as highlighted by the study, incorporating religious and spiritual counseling into support services can be beneficial. This could involve collaboration with religious leaders to provide solace and guidance to individuals, aligning with their faith-based coping strategies.

Encouraging open communication about miscarriage within communities can reduce stigma and provide comfort to those affected by the loss. Educational programs should be implemented to inform the public about the emotional and psychological impacts of miscarriage, emphasizing the importance of supportive dialogue and dispelling myths related to blame and fault.

Developing gender-sensitive approaches in support services can help address the different ways men and women experience and cope with miscarriage. Tailoring support to recognize these differences can ensure that both partners receive appropriate care and validation of their experiences.

Policy changes that recognize the emotional and psychological impacts of miscarriage should be made. Policies that mandate training for healthcare providers, funding for support services, and public education campaigns can enhance the support available to individuals and reduce the stigma surrounding miscarriage.

Further research focusing on recurrent miscarriages and miscarriage within other districts is highly recommended. Additionally, quantitative research is recommended to create a statistical understanding of the magnitude of the effect that miscarriages have.

Given the impact miscarriage has on the psychological, emotional, and social wellbeing of both women and men, psychotherapy and counseling services should be available to all individuals following the experience. This can be done on an individual, couple, or support group level to facilitate wellbeing.

## Conclusion

This article has discussed the findings from in-depth interviews with women and men who have experienced miscarriage(s), highlighting the complex experiences, coping strategies, and relationship dynamics involved. The themes identified provide a nuanced understanding of the lived experiences of miscarriage, the coping strategies employed by women and men, and the changes in relationship dynamics post-miscarriage. From the discussion, it is evident that there are diverse experiences ranging from emotional, psychological, to social, and individuals cope in different ways that may be predetermined by their religious affiliations, societal expectations, and other factors. Additionally, miscarriages have negative and positive effects on relationship dynamics, with the former being more predominant. Therefore, the discussion underscores the need for comprehensive and culturally sensitive support from society and institutions for individuals navigating the aftermath of miscarriage.

## Data Availability

The datasets presented in this study can be found in online repositories. The names of the repository/repositories and accession number(s) can be found at: https://share.google/l53gdB9aaHl1f7quh.
